# Paradigm Shift in Materials for Skull Reconstruction Facilitated by Science and Technological Integration

**DOI:** 10.7759/cureus.28731

**Published:** 2022-09-03

**Authors:** Arushi Beri, Sweta G Pisulkar, Akansha V Bansod, Chinmayee Dahihandekar

**Affiliations:** 1 Prosthodontics, Sharad Pawar Dental College and Hospital, Datta Meghe Institute of Medical Sciences, Wardha, IND; 2 Prosthodontics and Crown & Bridge, Sharad Pawar Dental College and Hospital, Datta Meghe Institute of Medical Sciences, Wardha, IND

**Keywords:** synthetic materials, xenografts, allografts, autologous grafts, cranioplasty

## Abstract

The surgical repair of a bone deficiency in the skull caused by a prior procedure or accident is known as cranioplasty. There are various types of cranioplasties, but the majority entail raising the scalp and reshaping the skull using either the original piece of bone from the skull or a specially molded graft created from Titanium (plate or mesh), artificial bone in place of, a stable biomaterial (prefabricated customized implant to match the exact contour and shape of the skull). Cranioplasty, one of the oldest surgical treatments for cranial abnormalities, has undergone several changes throughout the years to discover the best material to improve patient outcomes. Various materials have been utilized in cranioplasty throughout history. As biomedical technology progresses, surgeons will have access to new materials. There is still no agreement on the optimum material, and research into biologic and nonbiologic alternatives is ongoing in the hopes of finding the finest reconstruction material. The materials and techniques used in cranioplasty are covered in this article.

## Introduction and background

Cranial surgery can be traced back to 7000 B.C., according to archaeological data. The Incans, Britons, North Africans, and Polynesians were among the ancient civilizations that employed cranioplasty to correct cranial abnormalities. A frontal deformity on the left side of a Peruvian skull dating from 2000 BC was covered with a 1-mm-thick gold plate, which is a noteworthy example of obsolete cranioplasty [1]. During this time, the material used for cranioplasty was determined by the patient's financial situation. Gourds were used for the replacement of cranial defects in the common man, while precious metals were used for nobility. Fallopius proposed the use of bone in cranial defects if the Dura remained intact in the 14^th^ century [2]. The bone was replaced with a gold plate if there was a fracture of the dura [3]. Craniofacial deformities can be caused by multiple conditions like malignancy, injuries due to road traffic accidents, other trauma, infections, and genetically inherited diseases. It has the potential to hamper the quality of life by interfering with fundamental activities of patients such as communication, breathing, feeding, and aesthetics. Maxillofacial rehabilitation of the defects may possess a great challenge to clinicians due to defect complexity. A maxillofacial prosthesis is used to restore the deformed part of the cranium and/or face such as the nose, auricle, orbit, and surrounding tissues, with the primary goal of increasing the patient's quality of life by rehabilitating oral functions such as speech, swallowing, and mastication [4]. The neurosurgeon and prosthodontists are the members of the management team for patients with craniomaxillofacial defects. The patients experience psychological distress as a result of these problems and present to the doctor with several complaints related to the condition, such as a sense of helplessness, abandonment, and impairment [5]. The advancement of materials utilized in cranioplasty, including autologous grafts, allografts, xenografts, and a wide spectrum of synthetic materials, is discussed in this review article.

## Review

Xenograft

According to historical data, clinicians have attempted to correct cranial abnormalities by implanting animal tissue. Van Meekeren performed the first canine-to-human bone union in 1668 [6]. Humans have received bone grafts from dogs, apes, geese, rabbits, calves, and hawks. Better outcomes with autografts and other bone replacement therapies in the lab reduced the credibility of xenograft research. The goal of a study by Hakan Develioglu et al. was to examine the effects of a xenograft (Unilab Surgibone® [Mississauga, Ontario, Canada]) on rats with parietal bone deficiencies that were artificially produced. In order to do this, 14 rats were used in the current investigation, and 5-mm-diameter lesions were made on the parietal bone in each of them. While the left sites served as a control, the right defect sites received the xenograft material. The rats were killed after 30 days, and tissue samples were taken from the skull defects. In contrast to the control area's dense collagenous tissue, the implantation site's xenograft particles were encircled by a fibrous tissue layer. The examined xenograft appeared biocompatible and based on the results, it may be suggested as a suitable material for filling osseous abnormalities [7].

Allograft

In 1915, Morestin performed cranioplasty using cadaver cartilage. Cartilage was supposed to perform brilliantly since it molded well to fill flaws and was infection resistant [7]. Later, it was revealed that the cartilage was insufficient and that there had been no major calcification. Sicard and Dambrin experimented with cadaveric skulls in various ways in 1917. Sodium carbonate, xylol, liquor, and ether were used to treat the heat-sanitized resected bone [8]. Only the outside table remained after the thickness of the bone was reduced to the point where it could be punctured for use. Cadaveric skull allografts have become a dismal alternative for cranioplasty due to high rates of contamination and bone loss.

Autologous bone graft

Autologous bone graft replacement uses a recently removed bone graft for the correction of cranial deformity. The amount of foreign material that enters the body is reduced since the graft is instantly accepted by the host and incorporated into the cranium [9]. The ileum, ribs, sternum, and scapula were among the bones taken. The Müller-König procedure, which employed swinging, flaps of nearby tissue like skin, periosteum, and external table to correct cranial abnormalities, made the skull famous for autologous bone graft [10]. When cranial bone grafts are separated, donor site repair is easier, resulting in lower donor site morbidity. An autologous split-thickness bone transplant is the preferred graft for craniofacial restoration in children. A common problem in children is the resorption of the bone flap, which leads to underlying disintegration [11].

Synthetic materials

Synthetic materials are becoming more popular for cosmetic contouring of cranial defects because they eliminate bone resorption, contamination, donor site morbidity, and decreased strength and pliability and use of an antibiotic coating, as a result, engineered materials are becoming increasingly popular for cosmetic contouring [12,13]. Although autologous bone grafts are preferable due to their cure rate, reduced cost, and patient integration. The materials used in cranioplasty are compared in Table 1. 

**Table 1:** Materials used in cranioplasty [14].

Material	Advantages	Disadvantages
Autologous bone	The host accepts it, and it has a low fracture rate.	Bone resorption and infection.
Hydroxyapatite	It is anti-inflammatory, has a strong chemical bond with bone, and has excellent cosmesis and contouring capabilities.	Consider low tensile strength, brittleness, infection, fragmentation, and a lack of osteointegration.
Titanium mesh	This material's benefits include being non-inflammatory, non-corrosive, sturdy, malleable, low infection rate, and good cosmesis.	Expensive, having artefacts in the imaging.
Alumina ceramics	Low infectivity, hardness, chemical stability, and tissue compatibility	Expensive and easily broken.
Polyether Ether Ketone implant	It's radiolucent, chemically inert, sturdy, elastic, doesn't generate imaging artefacts, is pleasant to wear, and doesn't conduct heat.	All aspects to consider include cost, the necessity for extra 3D planning and imaging, the difficulty of attaching to other materials, and infection.
Poly Methylmethacrylate	Ease of use, excellent cosmesis, low cost, strength and durability.	Infection, plate fracture, exothermic reaction, inflammatory reaction.
Cortoss	High strength.	Exothermic reaction.
Calcium phosphate bone cement	Osteoconductive, useful for difficult to reach defects, no inflammatory reaction	Brittle, difficult to contour.

**Figure 1:**
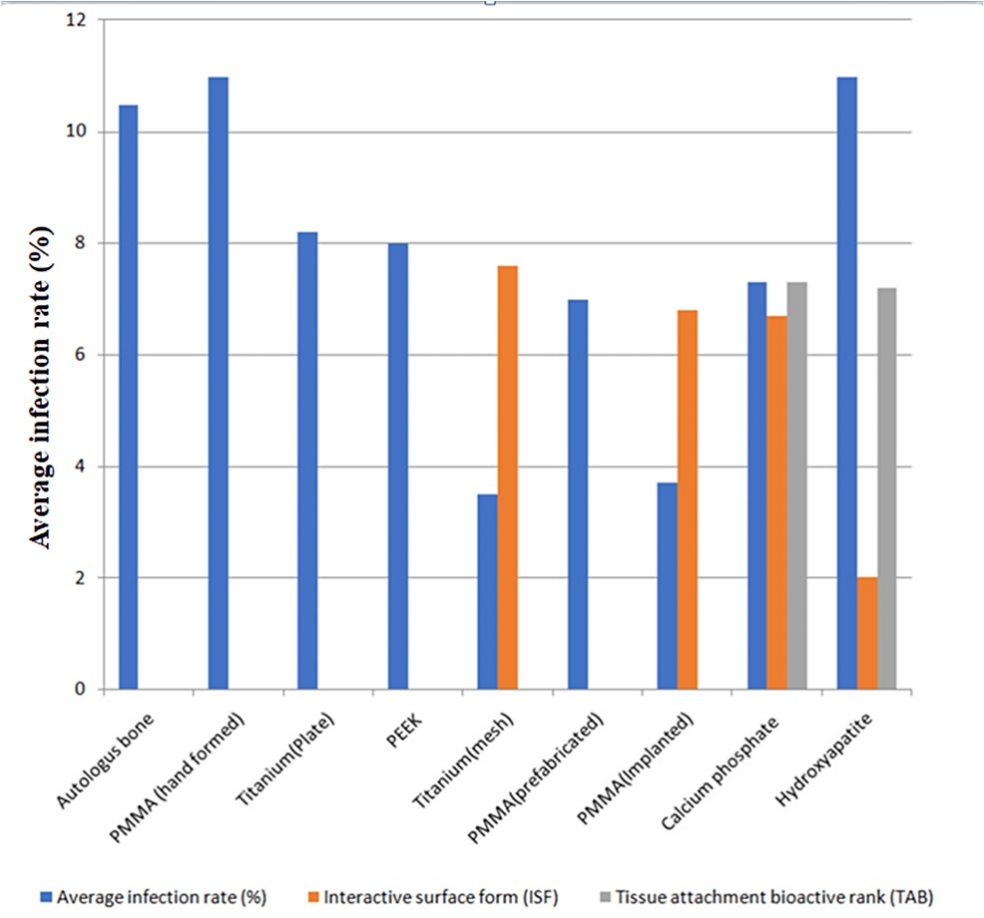
Cranioplasty/craniofacial implant material comparison: Surface form and tissue attachment associated infection risk [15].

Figure 1 shows cranioplasty/craniofacial implant material comparison: Surface form and tissue attachment associated infection risk [15].

Metals

Metals have been used since antiquity for cranioplasty. Metals have been investigated in a variety of ways due to their solidity, ability to be cleaned, and moldability [16]. In the late 1800s, aluminum was the most widely used metal, but it was prone to infection, irritates surrounding tissues, produces convulsions, and degrades slowly [17]. Gold did not demonstrate tissue reactivity and is not used because of its high cost and brittle nature. Sebileau offered silver in 1903, but it was rejected because silver oxide reacts with the scalp's surrounding tissues and discolors it [18]. Silver was also extremely fragile and vulnerable to damage. Tantalum was a rare and expensive metal that was difficult to come by. It has a high thermal conductivity, which causes headaches that are temperature dependent. Eventually, the emphasis shifted from metals to acrylics [19].

Methyl methacrylate (MMA)

Methacrylate (MMA), a polymerized acrylic acid ester, has the strength of bone. Furthermore, MMA outperforms hydroxyapatite in terms of compression and stress resistance. The dura mater has been found to cling to acrylic without causing tissue injury [20]. Acrylic bone cements (ABCs) consisting of polymethyl methacrylate (PMMA), which functions as a glue between the implant and the bone and is categorized as an inert substance, are used to secure most prostheses because of its intrinsic features, such as biocompatibility, antibacterial activity, bioactivity, regulated breakdown rate, and osteoconductive and osteoinductive activities, chitosan is a polymer of great interest for biomedical applications and implants.

In biomedicine, graphene oxide (GO) nanoparticles are used. The mechanical reinforcement of GO can help biomaterials like chitosan, polycaprolactone, and polylactic acid. GO could be utilized to treat resistant bacterial infections because it has a natural antibacterial activity. Because of its low cytotoxicity, GO may be a good alternative for bone tissue regeneration, bone cements, and cartilage regeneration in orthopedic applications [21].

Hydroxyapatite

Metal and other artificial materials have the drawback of being difficult to mold to the natural skull form; hydroxyapatite has been offered as a solution to this problem. Hydroxyapatite is a calcium phosphate molecule present in the bone that may be chemically synthesized into a ceramic [22]. To make a more stable prosthesis, hydroxyapatite can be combined with a titanium network [23]. The foreign body causes a modest reaction, and the chemical connection to the bone is minimal. The use of hydroxyapatite has been restricted because of its weak nature, poor flexibility, and high contamination rates [24].

Titanium meshwork and titanium lattice

To reinforce the prosthesis, it can be utilized alone or in combination with other produced components. Titanium is a metal alloy that combines pliability and overall strength [25]. Titanium is a non-corrosive, non-inflammatory metal ideal for restorative applications. Computer-assisted 3D modeling can be used to create titanium network inserts with excellent cosmesis even when severe cranial anomalies are present [26].

Alumina ceramics

Because of their endurance and cosmetic features, alumina ceramics have been a popular cranioplasty alternative in the previous decade. These ceramics are long-lasting, chemically inert, and have a tissue consistency similar to acrylics [27]. The radiopaque ceramic substance is made with yttrium. Changed ceramics are extremely expensive, require performing, and are prone to cracking, to name a few disadvantages.

Implants made of PEEK (polyetheretheretherketone)

These implants are nearly identical to the cortical bone in terms of strength, thickness, and flexibility, and they can be fused precisely inside the deformity without the use of miniplates. Since advances in 3D printing, PEEK inserts are commonly used. These personalized implants could be designed using computer-assisted 3D modeling [28]. The following are some of the most significant advantages of employing PEEK inserts: PEEK inserts are made of a thinner, lighter material than metallic implants, and because they are nonmagnetic and transparent to X-rays, they do not cause artifacts on computed tomography (CT) or magnetic resonance imaging (MRI) scans. They do not conduct heat, which can have a substantial impact on the brain, unlike metallic implants [29]. Steam or gamma rays can be used to disinfect the semicrystalline radiolucent polymer PEEK. They are expensive and require osteointegration qualities, notwithstanding their benefits.

Recent advancements

Cortoss (Orthovita, Malvern, PA, USA)

Cortoss^TM^, a novel synthetic bone void filler, is made of bis-glycidyl-methylmethacrylate, bisphenol (a polyethylene glycol dimethyl acrylate), triethylene glycol dimethyl acrylate monomer, and bioactive glass-ceramic [30]. It comes with mixing tips and is packaged in a two-lumen cartridge. Polymerization begins when the composite has been expressed through these locations, and the material is ready for usage. Cortoss^TM^ is a non-volatile monomer that polymerizes into a three-dimensional network to prevent leakage.

Endoscopic cranioplasty

Endoscopic technology and procedures have progressed, allowing surgeons to do a cranioplasty with little invasiveness. Materials such as acrylic, hydroxyapatite, and choral can be transferred through small wounds using endoscopic equipment [31]. Using a 3D digital printing model for cranial surgery to achieve the desired shape, a computer-aided design system and direct computer-aided manufacture are employed in the pre-operative production (i.e., prefabrication) of a cranioplasty prosthesis. This approach makes use of commonly available 3D CT data [32]. The cranial defect boundary can be seen on a 3D head CT-scan image of the skull surface. The patient's skull surface image is then fitted with a right-to-left mirrored image or an average 3D skull surface template image. The defect margin is stitched to the previously isolated defect margin after the region surrounding the problem is trimmed away. The defect-filling surface is then tapered and 3D printed. After that, the implant model is 3D printed and then molded into a biocompatible substance [33]. Because PEEK implants and titanium mesh can be 3D manufactured to fit the demands of each patient, they are used [34,35].

## Conclusions

Although metals have been used for cranioplasty since antiquity, autologous bone grafts are preferred because they prevent foreign components from entering the body and allow the bone flap to integrate back into the skull more quickly. Despite these benefits, the risk of infection, absorption, and weakness has led to an increase in synthetic materials used. Although there is no ideal cranioplasty material, empirical studies show that materials that are robust, infection-resistant, radiolucent, affordable, easy to work with, and capable of reintegrating with a patient's craniotomy defect will yield the best results for these patients.
